# A Small RNA-Based Immune System Defends Germ Cells against Mobile Genetic Elements

**DOI:** 10.1155/2016/7595791

**Published:** 2015-11-22

**Authors:** Astrid D. Haase

**Affiliations:** Laboratory of Cell and Molecular Biology, National Institute of Diabetes and Digestive and Kidney Diseases, National Institutes of Health, Bethesda, MD 20892, USA

## Abstract

Transposons are mobile genetic elements that threaten the survival of species by destabilizing the germline genomes. Limiting the spread of these selfish elements is imperative. Germ cells employ specialized small regulatory RNA pathways to restrain transposon activity. PIWI proteins and Piwi-interacting RNAs (piRNAs) silence transposons at the transcriptional and posttranscriptional level with loss-of-function mutant animals universally exhibiting sterility often associated with germ cell defects. This short review aims to illustrate basic strategies of piRNA-guided defense against transposons. Mechanisms of piRNA silencing are most readily studied in *Drosophila melanogaster*, which serves as a model to delineate molecular concepts and as a reference for mammalian piRNA systems. PiRNA pathways utilize two major strategies to handle the challenges of transposon control: (1) the hard-wired molecular memory of prior transpositions enables recognition of mobile genetic elements and discriminates transposons from host genes; (2) a feed-forward adaptation mechanism shapes piRNA populations to selectively combat the immediate threat of transposon transcripts. In flies, maternally contributed PIWI-piRNA complexes bolster both of these lines of defense and ensure transgenerational immunity. While recent studies have provided a conceptual framework of what could be viewed as an ancient immune system, we are just beginning to appreciate its many molecular innovations.

## 1. Mobile Genetic Elements Threaten Genomic Integrity

Transposons are mobile genetic elements that can move into novel locations within the genome. These genetic parasites have long colonized large portions of all eukaryotic genomes [1]. Transposons are classified based on their movement strategies: retrotransposons move through a “copy and paste” mechanism, involving reverse transcription of initial RNA copies and consecutive insertion into novel genomic regions. This mechanism does not alter the original genomic insertion and results in amplification of the element. In contrast, DNA transposons employ a “cut and paste” mechanism. Transposition to a new genomic location leaves a gap at the donor site that upon repair either results in restoration or in loss of the original insertion [2–7]. While the journey of transposons is often neutral to the host, novel insertions can cause severe damage, or, in rare cases, beneficial changes.

During evolution, host genomes accumulated scars while eliminating deleterious insertions and selecting for advantageous mutations. Above all, genomes devised mechanisms to repress transposon activity. Germline genomes constitute a crucial battleground for the arms race between transposons and their hosts. To ensure vertical transfer and amplification of a mobile element, transposition has to take place in germ cells. Transposons continuously adapt to thrive in this particular environment and germ cells in turn have devised specialized strategies to guard their genomic integrity and thus the continuation of a species [6, 8]. With defense mechanisms in place, host genomes seem to have come to equilibrium with their parasites. Most current transposon insertions are defunct, representing defeated fragments rather than powerful insurgents [1, 9].

Tamed transposons become part of the host's evolutionary toolkit and serve as a rich source of coding and noncoding sequences that allow for genetic innovation [3, 4]. A prominent example of such domestication is Telomere reverse transcriptase, which probably evolved form an ancient retrotransposon [10]. In* Drosophila melanogaster*, retroelements themselves have colonized telomeric regions and directly maintain chromosomal ends without the need for an active telomerase [11, 12]. In addition to domestication of coding sequences, transposon fragments have shaped gene regulatory networks by providing an arsenal of noncoding building blocks [2, 13, 14]. Despite these important positive contributions that have been selected for during evolution, transposons are intrinsically selfish and their activity must be tamed or it will threaten the integrity of host genomes.

Controlling the activity of transposable elements presents two major challenges: (1) to recognize transposons as “nonself” and to (2) mount an efficient defense selectively against active elements. Recognizing transposons as “nonself”, thereby discriminating them from host genes is not trivial. Transposons have become integral parts of our genomes and their mastery of camouflage enables them to hijack host machineries for transcription and translation. Additionally, their sequence diversity prevents recognition of specific motifs and their many mechanisms to transpose do not share vulnerable cofactors. In addition to direct genomic damage though transposition, transcription of certain elements can be toxic to the cell through the immense amount of produced RNA transcripts [15]. Adapting to the immediate threat of active transposons requires a prompt and specific defense. Small noncoding RNAs rise to the challenges posed by mobile genomic parasites [16–19]. Specialized small RNA pathways recognize transposons through the molecular memory of individual mobile sequences and have devised an elegant adaptive response against active elements.

## 2. Small RNA Pathways Are Prevalent in Eukaryotes

RNA interference (RNAi) was first observed as transgene cosuppression in plants [20]. Subsequent studies in* Caenorhabditis elegans *identified double-stranded RNA as the trigger of homology dependent gene silencing with corresponding small RNA products (~20–30 nt in length) serving as executive guides [21, 22]. Conserved small RNA pathways play crucial roles in development and disease [23–27]. These pathways can silence expression of target genes at transcriptional (i.e., recruiting histone and DNA methylation) or posttranscriptional level (i.e., promoting RNA degradation, inhibiting translation) [28–32]. At the heart of all RNA silencing pathways resides an RNA induced silencing complex (RISC), which in essence consists of a small noncoding RNA and its associated Argonaute protein partner [33, 34]. Within RISC, the small RNA determines target specificity by complementary base pairing, while its Argonaute partner governs effector mechanisms. Argonaute proteins are defined by a PAZ (Piwi-Argonaute-Zwille), a MID (middle), and a PIWI domain. The PAZ and MID domains specifically interact with the small RNA partner, anchoring its 3′ and 5′ termini, respectively [35]. The PIWI domain structurally resembles an RNase H fold and harbors RNA-guided endoribonuclease activity [36]. Phylogenetically Argonaute proteins segregate into two conserved subfamilies: the Ago-clade, similar to* Arabidopsis thaliana *AGO1, and the PIWI-clade, named after* Drosophila piwi* (P-element induced wimpy testis [37]) [38]. Members of the Ago-clade are ubiquitously expressed and associate with microRNAs (miRNAs) and small interfering RNAs (siRNAs). In contrast, the PIWI-clade is mostly restricted to germ cells in animals. PIWI proteins associate with a less well-understood class of small RNAs, piRNAs. PIWI-piRNA complexes silence transposons at the transcriptional and posttranscriptional level to guard the integrity of germline genomes [39–41].

## 3. Strategies of piRNA Pathways to Guard Genomic Integrity in* Drosophila melanogaster*

**Figure 1:**
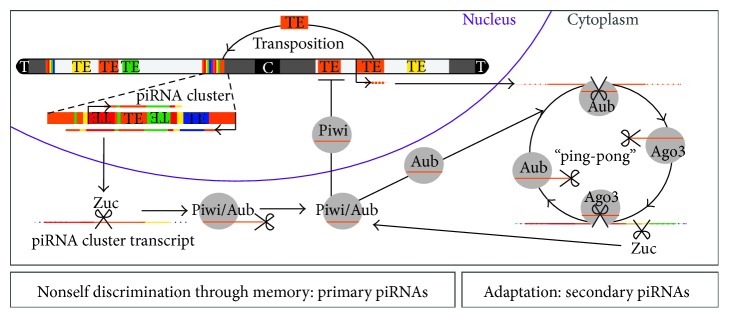
Mechanisms of “nonself” discrimination and adaptation in piRNA-guided defense against transposons in* Drosophila*. PiRNA clusters are genomic intervals that accumulate defunct fragments of transposable elements (TE) as a record of prior mobile activity. (1) Cluster regions are unidirectionally or bidirectionally transcribed and give rise to long presumably single stranded transcripts. PiRNA cluster transcripts are specifically processed into primary piRNAs by the consecutive action of at least two nucleases. The endonuclease Zucchini (Zuc) generates the 5′ terminus of a primary piRNA that is loaded into Piwi or Aubergine (Aub) and then trimmed by a 3′-5′ exonuclease. Piwi-piRNA complexes enter the nucleus and recruit chromatin modifying enzymes to silence transposon loci. In contrast, Aub-piRNA complexes initiate an adaptive response against transposon transcripts in cytoplasmic germ granules. (2) Aub and Ago3 engage in a feed-forward adaptation mechanism—the “ping-pong” cycle—that degrades transposon transcripts and concomitantly produces secondary piRNAs to selectively enhance the response against active elements. Through a feedback mechanism secondary piRNAs can initiate further Zuc-dependent piRNAs that are loaded into Piwi and Aub (Centromere (C), Telomere (T)).

PiRNAs greatly differ from miRNAs and siRNAs in their biogenesis, Argonaute protein partners, and expression patterns [42, 43]. In contrast to miRNAs and siRNAs, biogenesis of piRNAs does not depend on the RNase III enzyme Dicer [44]. PiRNAs are thought to be processed from long single stranded precursors that get parsed into an army of small RNAs with the potential to cooperatively target individual transposons. Owing to their sequence diversity and the lack of unique molecular characteristics, piRNAs are best defined by their physical and functional association with PIWI proteins [44–48]. Mechanisms of piRNA biogenesis and effector functions are best understood in the female germline of* Drosophila melanogaster*. It should be noted that piRNA pathways also operate in the male germline of flies, but their mechanisms are less well characterized [49, 50]. Three PIWI proteins, Piwi, Aubergine (Aub), and Ago3, are expressed during oogenesis in* Drosophila* and associate with piRNAs to form piRNA-silencing complexes (piRISCs) (Figure 1). Aub- and Ago3-piRISCs reside in the cytoplasm to degrade transposon transcripts [45, 51, 52]. In contrast, Piwi-piRISC localizes to the nucleus and induces transcriptional silencing at transposon loci [53–55]. Loss of either* PIWI *gene results in sterility of the animals, presumably as a consequence of uncontrolled transposon activity in the germline [37, 56–60].

PiRNAs can be grouped into two classes that represent molecular answers to the two major challenges of transposon control: “nonself” discrimination and selective adaptation to an immediate threat. “Primary piRNAs” and their generative loci, so-called piRNA clusters, achieve “nonself” recognition through extensive genetic memory of individual transpositions. “Secondary piRNAs” defend against transposon activity through selective amplification of sequences that target transposon transcripts. Primary and secondary piRNAs require distinct processing enzymes and cofactors, but they function collaboratively to protect the integrity of germline genomes [32, 61–63].

### 3.1. Memory of Previous Transpositions Enables “Nonself” Recognition Guided by Primary piRNAs

Most primary piRNAs originate from a limited number of discrete genomic regions termed piRNA clusters that are defined as genomic intervals with a high density of uniquely mapping piRNAs [45] (Figure 1). Some of these ~140 genomic regions were previously linked to transposon control [64–67]. PiRNA clusters act as transposon traps, accumulating numerous transposon insertions over time and retaining a collection of densely packed defunct fragments. Insertion of novel sequences into piRNA clusters adds information to the repository, marks corresponding elements as “nonself” and confers resistance in trans [68, 69]. piRNA clusters are a fossil record of transposition activity, reflecting the mobile heritage of genomes and providing a molecular database for “nonself” recognition. How piRNA clusters are formed and how their transcripts are specifically marked for processing into piRNAs are major outstanding questions in the field.

Most piRNA clusters contain transposon insertions in mixed orientation and generate transcripts from both genomic strands. Transcriptional regulation of these dual-strand piRNA clusters differs from genic transcription in respect to interpretation of chromatin marks and cotranscriptional processing of nascent transcripts. In contrast to active genes, piRNA clusters preferentially reside in pericentric or subtelomeric regions that mark the boundaries of constitutive heterochromatin and euchromatin [70]. Cluster transcription requires recruitment of Rhino, a fast evolving heterochromatin protein 1 (HP1) family member, to trimethylated lysine 9 of histone H3 (H3K9me3), which otherwise typically marks silent genes [71–74]. Rhino associates with the adaptor protein Deadlock to recruit Cutoff, a homolog of the Rai1/Dom3Z decapping enzyme, which has lost its enzymatic activity. The Rhino-Deadlock-Cutoff complex is suggested to protect the 5′ end of nascent cluster transcripts and to suppress both canonical splicing and transcriptional termination [74–78]. Moreover, Rhino recruits the RNA helicase UAP56 to specify cluster transcripts for transport to their processing site [78]. Using a positive feed-back mechanism, Piwi-piRNA complexes themselves maintain transcriptional activity at cluster loci through recruitment of H3K9me3 [79–81].

PiRNA clusters give rise to long single-stranded transcripts without known structural or sequence determinants or significant formation of double-stranded RNA. These cluster transcripts are processed into a large body of diverse small RNAs by the consecutive action of at least two nucleases. An endoribonuclease generates the 5′ monophosphorylated end of a new piRNA that is consecutively loaded into PIWI's MID pocket. Consecutively, the 3′ end of the PIWI-bound pre-piRNA is trimmed by a 3′ to 5′ exonuclease. The endonucleolytic activity that likely generates the 5′ terminus of a primary piRNA can be assigned to the conserved nuclease Zucchini (Zuc) [82–84]. In contrast, the identity of the 3′ trimming exonuclease remains elusive [85]. The final length of a mature piRNA is marked by 2′-O-methylation and likely represents a footprint of its associated PIWI protein [85–87]. Little is known about the initial processing of piRNA cluster transcripts. While primary processing intermediates or degradation fragments have been observed, the precise mechanisms remain obscure [88]. Further characterization of piRNA cluster transcripts and identification of the factors that specify transcripts for processing into piRNAs are required to understand how hardwired memory of past transpositions is parsed into small RNA guides for “nonself” recognition.

### 3.2. An Adaptive Mechanism That Produces Secondary piRNAs Defends Selectively against Mobile Elements That Have Evaded Silencing at the Site of Transcription

PiRNAs engage in a feed-forward adaptive response to specifically eliminate cytoplasmic transposon transcripts and reinforce piRNA production. This robust strategy relies on removal of transposon transcripts through piRNA-guided cleavage and simultaneous production of select secondary piRNAs [45, 51, 52]. In contrast to primary piRNAs, which represent all indexed transposons irrespective of their transcript abundance, secondary piRNAs shape the overall piRNA pool toward recognition of active elements. Biogenesis of secondary piRNAs relies on the nuclease activity of two PIWI proteins, Aub and Ago3, that collaborate in the so-called “ping-pong” cycle [45, 52]. Processing of secondary piRNAs is believed to be triggered by either primary or maternally contributed Aub-piRNAs. These piRNAs guide Aub to cleave complementary transposon transcripts. Target RNA cleavage by Aub generates the 5′ end of a new piRNA that is loaded into Ago3. Because Ago3-piRNAs originate from transposon transcripts themselves, they carry selective information about these potentially harmful active elements. Ago3-piRNAs in turn guide cleavage of complementary stretches within cluster transcripts to generate additional Aub-piRNAs and complete the “ping-pong” cycle [57] (Figure 1). Multiple rounds of selection alternate between transposon and cluster transcripts as substrates for secondary piRNA production and mold piRNA populations towards preferentially target active elements. During “ping-pong,” transposon-triggered processing of cluster transcripts selects for sequences that are genetically determined as “nonself.” This mechanism could act as a protective measure to prevent amplification of piRNAs that accidentally target host genes and thus could cause autoaggression, analogous to the phenomena of autoimmunity.

Coordination of this elegant adaptation mechanism is achieved in specialized perinuclear germ granules called Nuage [89]. Members of the Tudor protein family are key-components of these germ granules and play crucial roles in piRNA biology. Tudor proteins are defined by the presence of one or multiple Tudor domains that recognize methylated Arginine residues and facilitate protein interactions [90]. PIWI proteins are methylated at N-terminal Arginines and various Tudor proteins regulate interactions between PIWI proteins and cofactors to ensure efficient heterotypic processing of secondary piRNAs [91]. Dynamic orchestration of these interactions requires periodic remodeling of PIWI-RNA complexes by the DEAD box helicase Vasa, which chaperones the transfer of piRNA intermediates between PIWI “ping-pong” partners [92, 93].

Additionally, secondary piRNAs have the potential to trigger Zucchini-dependent processing of adjacent piRNA cluster regions into piRNAs that are loaded into Aub and Piwi. This feedback mechanism not only results in diversification of piRNA sequences, but also enables transmission of information from the adaptive defense to the transcriptional silencing machinery [94–96]. Additional genes involved in piRNA pathways emerged from genome-wide screens and further characterization of these factors will add to our mechanistic understanding of this elaborate small RNA based immunity [97–99].

### 3.3. Maternally Inherited PIWI-piRNA Complexes Bolster Trans-Generational Immunity

Genetic observations have long suggested the role for a maternally contributed factor in transposon control in* Drosophila*. Crosses of males carrying a specific transposon to females naïve to this element result in a loss of germ cells and sterility in the offspring. Interestingly, despite genetic identity, the reciprocal crosses do not exhibit defects in germ cell development. Germ cell defects in the dysgenic progeny are associated with mutations and chromosomal rearrangements attributed to unleashed transposon activity [100–105]. This phenomenon of hybrid dysgenesis suggests the requirement of nongenetic, maternally contributed factors in transposon defense [106, 107]. The nature of these factors remained mysterious until PIWI-piRNA complexes were identified as molecular determinants of maternal immunity [81]. Maternally contributed PIWI-piRNA complexes transmit immunity in two ways: (1) they reinforce memory through recruitment of H3K9me3 to piRNA clusters, thus bolstering noncanonical transcription at these loci, and (2) they directly initiate adaptive processing of secondary piRNAs through the “ping-pong” cycle [55, 80, 81, 108, 109].

## 4. Small RNA Pathways Guard Genomic Stability in Mammalian Germ Cells

Transposon sequences and active transposon families vary significantly between different species [110]. Considering coevolution between transposons and the host's defense system, adaptive variation in molecular mechanisms is expected. Yet, principles of RNA interference have seemingly proven efficient for defense. Like flies, mouse piRNA pathways employ three PIWI-like (PWIL) proteins: Piwil1/Miwi, Piwil2/Mili, and Piwil4/Miwi2 [46, 47, 111]. Removal of either PIWI gene results in germ cell defects accompanied by a burst in transposon activity in the male germline [112–115]. PiRNA pathways operate at two distinct stages during mouse spermatogenesis. In embryonic testes, Piwil2/Mili collaborates with Piwil4/Miwi2 to protect primordial germ cells. Similar to* Drosophila* piRNAs, mouse embryonic piRNAs mainly correspond to transposon sequences and engage in selective generation of secondary piRNAs in response to transposon transcripts. In contrast to flies, the “ping-pong” cycle in mouse involves the catalytic activity of only one PIWI partner, Piwil2/Mili [116]. Intra-Piwil2/Mili “ping-pong” generates secondary piRNAs that are loaded into Piwil2/Mili and Piwil4/Miwi2. Interaction with piRNAs licenses Piwil4/Miwi2 to transfer to the nucleus and silence transposon loci [113, 115, 116]. Recruitment of Piwil4/Miwi2-piRNA complexes results in histone and DNA methylation to full-length, potentially active transposon-loci [117, 118]. Interestingly, full-length transposons seem to evade a prior piRNA-independent wave of heterochromatinization in the embryonic gonad and require piRNA-guided control [117]. A second phase of piRNA silencing takes place in adult testis during entry into meiosis. Two cytoplasmic PIWI proteins, Piwil2/Mili and Piwil1/Miwi, are involved in this pathway. The functions and precise mechanisms of adult piRNAs remain enigmatic. Adult piRNAs mainly originate from relatively few intergenic clusters with little potential to target cellular transcripts but their own [111, 119]. Although this class of piRNAs is not enriched in transposon sequences and does not exhibit “ping-pong” activity, loss of Piwil1/Miwi, Piwil2/Mili, or either nuclease activity results in derepression of transposons [120, 121]. How adult piRNAs guide transposon silencing without obvious enrichment in complementary sequences remains elusive. Recent studies suggest a function for these piRNAs in posttranscriptional regulation beyond transposon silencing [122–124].

Curiously, while piRNAs are present in mouse oocytes, they are not required for fertility. In the female germline of mice transposon defense relies on endogenous siRNAs [125, 126]. These siRNAs originate from dsRNA precursors that are generated by the repetitive nature of transposon sequences or antisense transcription at corresponding loci. Long dsRNA substrates are processed into ~21-nt long siRNAs by the RNase III enzyme Dicer and guide Ago2 to slice transposon transcripts. Transposon loci produce piRNAs and siRNAs in a seemingly redundant fashion in the developing oocyte. However, siRNAs dominate piRNAs in abundance and function. It is puzzling how and why mouse oocytes chose siRNAs over piRNAs in transposons control. An unexpected recent finding shed some light on this paradox: mice express an oocyte-specific isoform of Dicer—Dicer^O^—that harbors enhanced activity on processing dsRNA into siRNAs [127]. Interestingly, transcription of Dicer^O^ is driven by an intronic transposon insertion. Deletion of this insertion causes loss of Dicer^O^ and female sterility and resembles a maternal Dicer null phenotype [128]. This observation emphasizes the tight relationship between transposons and their host even in establishing a transposon defense system. Transposon driven Dicer^O^ expression is specific to mice and rats. Thus, siRNA-mediated transposon silencing seems to be an exception rather than a rule in the mammalian germline, which prompts a reevaluation of piRNA function in the female germline of other mammals [129, 130].

## 5. Conclusion

Germ cells have devised small RNA mechanisms to master the challenges of transposon control. PiRNA clusters establish and maintain memory of “nonself” and generate mobile guides to induce an adaptive defense against active transposons. Maternally transmitted PIWI-piRNA complexes add an additional layer to transgenerational immunity against mobile genetic elements in flies. Over the past several years RNA-based immunity against genomic invaders has been discovered in all branches of life ranging from the CRISPR-Cas systems in bacteria to piRNAs in animals [18]. RNA-based strategies for “nonself” discrimination and adaptation have seemingly proven efficient to protect the integrity of genomes. Insights into the elegant mechanisms of these RNA-based immune systems will further our appreciation of the complex relationship between parasitic nucleic acids and their host genomes.
